# Invasive hydatidiform mole in the cervix

**DOI:** 10.11604/pamj.2018.29.27.10675

**Published:** 2018-01-12

**Authors:** Houssine Boufettal, Naïma Samouh

**Affiliations:** 1 Centre Hospitalier Universitaire Ibn Rochd, Faculté de Médecine et Pharmacie, Hassan II University of Casablanca, Casablanca, Maroc

**Keywords:** Invasive mole, cervix, gestational trophoblastic tumor, chemotherapy

## Image in medicine

A patient aged 43-years, multiparous consulted for uterine bleeding of average abundance. The examination revealed a burgeoning lesion of the cervix, which came from the uterine endocervix, measuring two centimeters. The uterus was increased in size measuring 88 mm long and 67 mm in anteroposterior diameter. There were no adnexal mass. Pelvic ultrasound showed a heterogeneous snowflake mass measuring 29 mm in anteroposterior diameter. Beta-h-CG (human chorionic gonadotrophin) quantitative plasma were highly increased to 854212 IU / ml. Histological study of aspirate objectified a complete hydatidiform mole. The staging featuring a thoraco-abdominopelvic CT scan, chest radiography, ultrasound abdomen and pelvis was normal. Pathological examination of the hysterectomy piece objectified an invasive mole to cervical and uterine location. A methotrexate-based agent chemotherapy was introduced. The evolution was marked by the gradual decline of the mass until it disappearance within four months. Plasma beta-h-CG had regressed and were normalized after two months of treatment. The outcome was favorable. With a follow-up of 24 months, no recurrence was noted.

**Figure 1:**
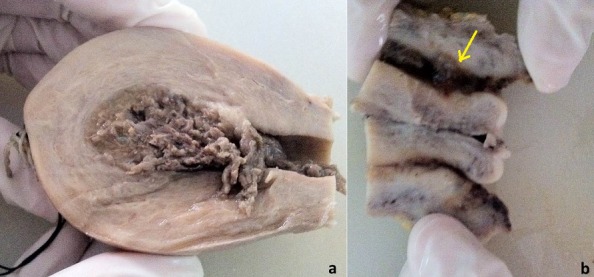
pathological examination of the hysterectomy piece objectified an invasive mole in cervical spine (arrow) and uterine

